# (*E*)-*N*-(4-{[1-(Prop-2-en-1-yl)-1*H*-1,2,3-triazol-4-yl]meth­oxy}benzyl­idene)morpholin-4-amine

**DOI:** 10.1107/S1600536814002827

**Published:** 2014-02-12

**Authors:** Mehmet Akkurt, Aliasghar Jarrahpour, Mehdi Mohammadi Chermahini, Pezhman Shiri, Namık Özdemir

**Affiliations:** a Department of Physics, Faculty of Sciences, Erciyes University, 38039 Kayseri, Turkey; b Department of Chemistry, College of Sciences, Shiraz University, 71454 Shiraz, Iran; c Department of Physics, Faculty of Arts and Sciences, Ondokuz Mayıs University, 55139 Samsun, Turkey

## Abstract

The asymmetric unit of the title compound, C_17_H_21_N_5_O_2_, contains two crystallographically independent mol­ecules, which are linked by a C—H⋯N hydrogen bond. The morpholine rings of both mol­ecules adopt distorted chair conformations. The dihedral angles between the triazole and benzene rings are 12.8 (3)° in the first independent molecule in which the –N=C– group between the morpholine and benzene rings is disordered [site-occupancy ratio = 0.576 (7):0.424 (7)] and 88.1 (2)° in the second independent mol­ecule. In the crystal, mol­ecules are linked by C—H⋯N hydrogen bonds along the [100] direction. In addition, one weak C—H⋯π inter­action and two weak π–π stacking inter­actions [centroid–centroid distances = 3.840 (3) and 3.823 (2) Å] between the triazole rings of adjacent mol­ecules are observed. The atoms of the terminal propenyl groups in both mol­ecules are disordered over two sets of sites [site-occupancy ratios = 0.691 (10):0.309 (10) and 0.705 (15):0.295 (15)].

## Related literature   

For the biological activity of triazole derivatives, see: Bringmann *et al.* (2004 ▶); Nelson *et al.* (2004 ▶); Nithinchandra *et al.* (2013 ▶); Sherement *et al.* (2004 ▶); Singh *et al.* (2012 ▶). For similar structures, see: Akkurt *et al.* (2013*a* ▶,*b* ▶). For puckering parameters, see: Cremer & Pople (1975 ▶).
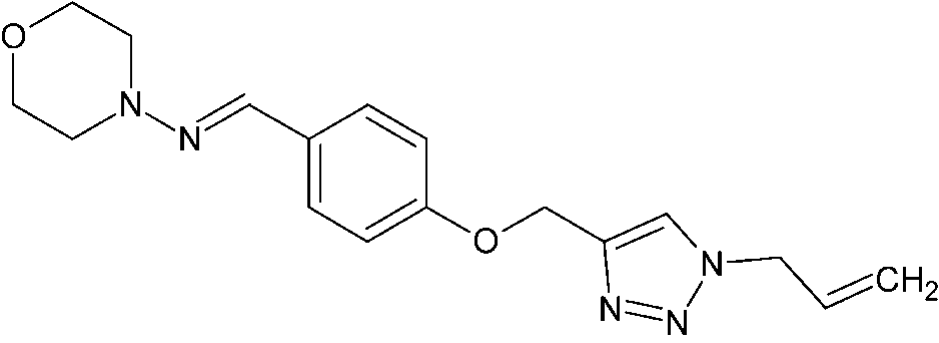


## Experimental   

### 

#### Crystal data  

C_17_H_21_N_5_O_2_
*M* *_r_* = 327.39
Triclinic, 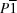

*a* = 10.5992 (8) Å
*b* = 11.6351 (10) Å
*c* = 14.8758 (13) Å
α = 95.811 (7)°
β = 100.724 (6)°
γ = 99.838 (6)°
*V* = 1759.1 (3) Å^3^
*Z* = 4
Mo *K*α radiation
μ = 0.08 mm^−1^
*T* = 296 K
0.58 × 0.34 × 0.13 mm

#### Data collection  

Stoe IPDS 2 diffractometer
Absorption correction: integration (*X-RED32*; Stoe & Cie, 2002 ▶) *T*_min_ = 0.968, *T*_max_ = 0.990
18485 measured reflections
6210 independent reflections
2458 reflections with *I* > 2σ(*I*)
*R*_int_ = 0.097

#### Refinement  

*R*[*F* ^2^ > 2σ(*F* ^2^)] = 0.080
*wR*(*F* ^2^) = 0.224
*S* = 0.90
6210 reflections
419 parameters
18 restraints
H-atom parameters constrained
Δρ_max_ = 0.59 e Å^−3^
Δρ_min_ = −0.25 e Å^−3^

### 

Data collection: *X-AREA* (Stoe & Cie, 2002 ▶); cell refinement: *X-AREA*; data reduction: *X-RED32* (Stoe & Cie, 2002 ▶); program(s) used to solve structure: *SHELXS2013* (Sheldrick, 2008 ▶); program(s) used to refine structure: *SHELXL2013* (Sheldrick, 2008 ▶); molecular graphics: *ORTEP-3 for Windows* (Farrugia, 2012 ▶); software used to prepare material for publication: *WinGX* (Farrugia, 2012 ▶).

## Supplementary Material

Crystal structure: contains datablock(s) global, I. DOI: 10.1107/S1600536814002827/hg5381sup1.cif

Structure factors: contains datablock(s) I. DOI: 10.1107/S1600536814002827/hg5381Isup2.hkl

Supporting information file. DOI: 10.1107/S1600536814002827/hg5381Isup3.cml

CCDC reference:

Additional supporting information:  crystallographic information; 3D view; checkCIF report

## Figures and Tables

**Table 1:** Hydrogen-bond geometry (Å, °)

*D*—H⋯*A*	*D*—H	H⋯*A*	*D*⋯*A*	*D*—H⋯*A*
C14—H14⋯N8	0.93	2.49	3.385 (6)	162
C15*A*—H15*B*⋯N3^i^	0.97	2.59	3.355 (18)	136
C31—H31⋯N3^ii^	0.93	2.54	3.323 (5)	141
C31—H31⋯N4^ii^	0.93	2.40	3.326 (5)	171
C24—H24⋯*Cg*3^iii^	0.93	2.92	3.710 (5)	144

Symmetry codes: (i) 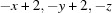; (ii) 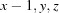; (iii) 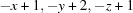.

**Fig. 1.**
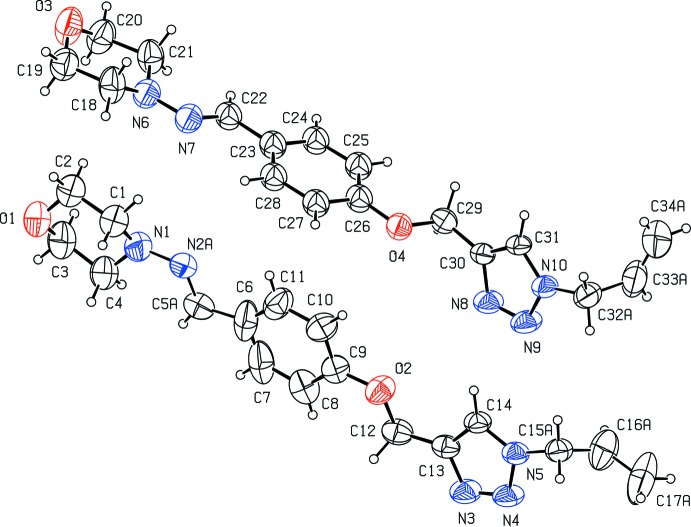
The title molecule (I) with the atom numbering scheme. Displacement ellipsoids for non-H atoms are drawn at the 30% probability level. Only major disorder component is shown.

**Fig. 2.**
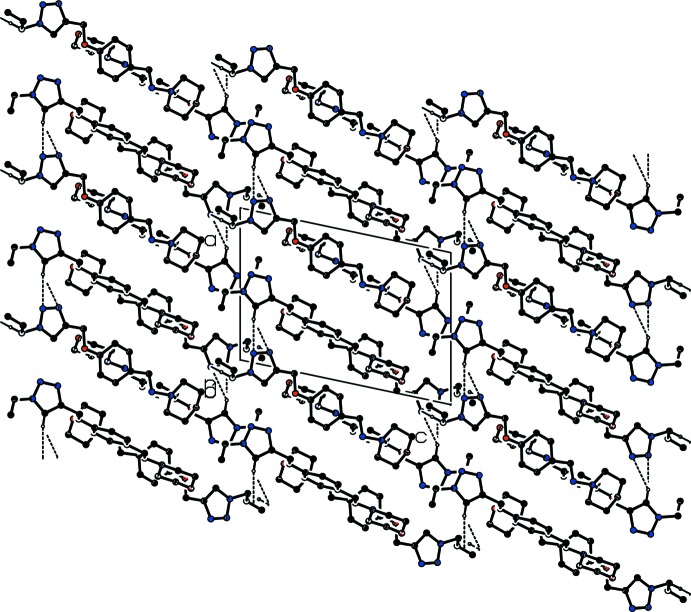
View of the hydrogen bonding and molecular packing of (I) along the b axis. H atoms involved in H bonding and only major disorder component of disorder are shown.

## supplementary crystallographic information

### 1. Comment

Schiff bases are present in various natural, semi-synthetic, and synthetic compounds and have been demonstrated to be essential for their biological activities (Bringmann *et al.*, 2004). Triazoles constitute an important class of heterocycles because of their varied biological activities (Singh *et al.*, 2012). 1,2,3-Triazoles are attractive constructs, because of their unique chemical properties and they find many applications in organic and medicinal chemistry (Nithinchandra *et al.*, 2013). They are found to be potent antimicrobial and antiviral agents (Sherement *et al.*, 2004). The morpholine moiety has been utilized extensively by the pharmaceutical industry in drug design, often because of the improvement in pharmacokinetic properties it can confer. The biological utility of molecules containing the morpholine moiety is wide-ranging (Nelson *et al.*, 2004). Therefore, compound (I), which has the triazole and the morpholine moieties in one molecule, has beeb synthesized and its X-ray studies is reported here.

Fig. 1 shows two crystallographically independent molecules in the asymmetric unit of the title compound. The morpholine rings of both molecules adopts distorted chair conformations [puckering parameters (Cremer & Pople, 1975) are Q_T_ = 0.415 (6) Å, θ = 4.2 (7)°, φ = 5(10)° and Q_T_ = 0.519 (6) Å, θ = 173.1 (7)°, φ = 169 (6)°, respectively]. The dihedral angles between the triazole and benzene rings of both molecules are 12.8 (3) and 88.1 (2) °, respectively. All bond lengths and bond angles in (I) are normal and comparable to those given for the similar compounds (Akkurt *et al.*, 2013*a*,*b*).

In the crystal, molecules are linked by C—H···N hydrogen bonds (Table 1 and Fig. 2) along the [100] direction. Furthermore, one weak C—H···π interaction and two weak π-π stacking interactions [*Cg*1···*Cg*1(2 - *x*, 2 - *y*, -*z*) = 3.840 (3) Å and *Cg*4···*Cg*4(1 - *x*, 2 - *y*, -*z*) = 3.823 (2) Å; where *Cg*1 and *Cg*4 are the centroids of the N3—N5/C13/C14 and N8—N10/C30/C31 triazole rings of the two molecules in the asymmetric unit, respectively] between the triazole rings of the adjacent molecules help to stabilize the crystal structure.

### 2. Experimental

Reaction of 4-((1-allyl-1*H*-1,2,3-triazol-4-yl)methoxy)benzaldehyde (1.00 mmol) with morpholin-4-amine (1.00 mmol) in refluxing ethanol gave the title compound. Recrystallization from ethanol gave colourless prisms in 75% yield. Mp: 410–412 K. IR (KBr, cm^-1^):1612 (C=N). ^1^H-NMR (250 MHz, CDCl_3_), δ (p.p.m.): 3.15 (CH_2_—N morpholine, t, 4H, J=5 Hz), 3.89 (CH_2_—O morpholine, t, 4H, J=5 Hz), 4.96 (d, 2H, J=5 Hz), 5.26 (s, 2H), 5.35 (m, 2H), 6.01 (m, 1H), 6.96 (aromatic H, d, 2H, J=10 Hz), 5.55 (aromatic H, d, 2H, J=7.5 Hz), 7.64 (H triazole, s, 1H), 7.75 (C=N, s, 1H). ^13^C-NMR (62.9 MHz, CDCl_3_), δ (p.p.m.): 52.3 (CH_2_—N morpholine), 52.7 (CH_2_—N), 62.0 (CH_2_—O morpholine), 66.1(CH_2_—O), 114.9–131.0 (aromatic carbons and C=C triazole), 159.2 (C=N).

### 3. Refinement

H atoms were located geometrically and were refined using a riding model with *U*_iso_(H) = 1.2*U*_eq_(C). The atoms of the terminal propenyl groups of the two molecules in the asymmetric unit are disordered over two sets of sites, with the site-occupancy ratios of 0.691 (10): 0.309 (10) and 0.705 (15): 0.295 (15), respectively. The –N2═C5– group between the morpholine and benzene rings of the one molecule is also disordered over two positions [site-occupancy ratio = 0.576 (7): 0.424 (7)]. The small proportion of reflections observed is a result of the rather poor quality of the very thin crystals obtained.

### Crystal data

**Table:**

C_17_H_21_N_5_O_2_	*Z* = 4
*M**_r_* = 327.39	*F*(000) = 696
Triclinic, *P*1	*D*_x_ = 1.236 Mg m^−^^3^
Hall symbol: -P 1	Mo *K*α radiation, λ = 0.71073 Å
*a* = 10.5992 (8) Å	Cell parameters from 12937 reflections
*b* = 11.6351 (10) Å	θ = 1.4–27.9°
*c* = 14.8758 (13) Å	µ = 0.08 mm^−^^1^
α = 95.811 (7)°	*T* = 296 K
β = 100.724 (6)°	Prism, colourless
γ = 99.838 (6)°	0.58 × 0.34 × 0.13 mm
*V* = 1759.1 (3) Å^3^	

### Data collection

**Table:**

Stoe IPDS 2 diffractometer	6210 independent reflections
Radiation source: sealed X-ray tube, 12 x 0.4 mm long-fine focus	2458 reflections with *I* > 2σ(*I*)
Plane graphite monochromator	*R*_int_ = 0.097
Detector resolution: 6.67 pixels mm^-1^	θ_max_ = 25.0°, θ_min_ = 1.4°
ω scans	*h* = −12→12
Absorption correction: integration (*X-RED32*; Stoe & Cie, 2002)	*k* = −13→13
*T*_min_ = 0.968, *T*_max_ = 0.990	*l* = −17→17
18485 measured reflections	

### Refinement

**Table:**

Refinement on *F*^2^	18 restraints
Least-squares matrix: full	Hydrogen site location: inferred from neighbouring sites
*R*[*F*^2^ > 2σ(*F*^2^)] = 0.080	H-atom parameters constrained
*wR*(*F*^2^) = 0.224	*w* = 1/[σ^2^(*F*_o_^2^) + (0.1115*P*)^2^] where *P* = (*F*_o_^2^ + 2*F*_c_^2^)/3
*S* = 0.90	(Δ/σ)_max_ < 0.001
6210 reflections	Δρ_max_ = 0.59 e Å^−^^3^
419 parameters	Δρ_min_ = −0.25 e Å^−^^3^

### Special details

**Table:**

Geometry. Bond distances, angles *etc*. have been calculated using the rounded fractional coordinates. All su's are estimated from the variances of the (full) variance-covariance matrix. The cell e.s.d.'s are taken into account in the estimation of distances, angles and torsion angles
Refinement. Refinement on *F*^2^ for ALL reflections except those flagged by the user for potential systematic errors. Weighted *R*-factors *wR* and all goodnesses of fit *S* are based on *F*^2^, conventional *R*-factors *R* are based on *F*, with *F* set to zero for negative *F*^2^. The observed criterion of *F*^2^ > σ(*F*^2^) is used only for calculating -*R*-factor-obs *etc*. and is not relevant to the choice of reflections for refinement. *R*-factors based on *F*^2^ are statistically about twice as large as those based on *F*, and *R*-factors based on ALL data will be even larger.

### Fractional atomic coordinates and isotropic or equivalent isotropic displacement parameters (Å^2^)

**Table:**

	*x*	*y*	*z*	*U*_iso_*/*U*_eq_	Occ. (<1)
O1	0.6237 (5)	0.4832 (3)	0.7915 (3)	0.1443 (19)	
O2	0.8543 (3)	0.9516 (3)	0.26209 (19)	0.1045 (14)	
N1	0.6676 (6)	0.6509 (4)	0.6716 (3)	0.126 (2)	
N2A	0.6643 (8)	0.6924 (8)	0.5811 (6)	0.094 (3)	0.576 (7)
N3	1.0870 (3)	1.0861 (4)	0.1389 (3)	0.1039 (16)	
N4	1.0574 (3)	1.1331 (3)	0.0639 (3)	0.1043 (16)	
N5	0.9289 (3)	1.1321 (3)	0.0491 (2)	0.0815 (12)	
C1	0.5505 (6)	0.5636 (6)	0.6529 (4)	0.133 (3)	
C2	0.5181 (6)	0.5210 (5)	0.7387 (4)	0.141 (3)	
C3	0.7331 (7)	0.5754 (5)	0.8149 (4)	0.148 (3)	
C4	0.7744 (6)	0.6183 (5)	0.7315 (4)	0.136 (3)	
C5A	0.7787 (8)	0.7292 (7)	0.5672 (6)	0.092 (3)	0.576 (7)
C6	0.7697 (11)	0.7785 (5)	0.4807 (4)	0.153 (4)	
C7	0.8949 (9)	0.8212 (5)	0.4742 (4)	0.151 (4)	
C8	0.9304 (5)	0.8784 (4)	0.4061 (3)	0.1147 (19)	
C9	0.8325 (5)	0.8945 (4)	0.3350 (3)	0.0918 (19)	
C10	0.7034 (6)	0.8523 (4)	0.3372 (3)	0.110 (2)	
C11	0.6738 (6)	0.7962 (5)	0.4088 (4)	0.126 (3)	
C12	0.9829 (4)	1.0002 (4)	0.2566 (3)	0.0962 (17)	
C13	0.9784 (4)	1.0560 (3)	0.1722 (3)	0.0797 (17)	
C14	0.8776 (4)	1.0853 (3)	0.1152 (3)	0.0802 (16)	
C15A	0.8680 (19)	1.1698 (15)	−0.0364 (9)	0.090 (3)	0.691 (10)
C16A	0.9190 (18)	1.2977 (16)	−0.0298 (7)	0.150 (5)	0.691 (10)
C17A	0.9566 (11)	1.3396 (12)	−0.1022 (8)	0.225 (7)	0.691 (10)
C5B	0.6811 (13)	0.7108 (13)	0.5400 (8)	0.092 (3)	0.424 (7)
C15B	0.855 (4)	1.187 (4)	−0.023 (2)	0.090 (3)	0.309 (10)
C16B	0.930 (5)	1.283 (4)	−0.065 (2)	0.150 (5)	0.309 (10)
C17B	0.853 (3)	1.359 (3)	−0.0446 (19)	0.225 (7)	0.309 (10)
N2B	0.7456 (10)	0.7084 (11)	0.6185 (7)	0.094 (3)	0.424 (7)
O3	0.0840 (5)	0.5145 (4)	0.7668 (3)	0.1466 (19)	
O4	0.3815 (3)	0.9503 (2)	0.23393 (16)	0.0879 (10)	
N6	0.1978 (4)	0.6094 (3)	0.6291 (3)	0.1035 (11)	
N7	0.2177 (4)	0.6515 (3)	0.5472 (2)	0.1035 (11)	
N8	0.5704 (3)	1.1125 (3)	0.1342 (3)	0.0960 (16)	
N9	0.5726 (3)	1.1426 (3)	0.0521 (3)	0.1002 (16)	
N10	0.4507 (3)	1.1526 (3)	0.0151 (2)	0.0797 (12)	
C18	0.1177 (6)	0.4959 (4)	0.6103 (3)	0.123 (2)	
C19	0.1057 (6)	0.4407 (5)	0.6941 (4)	0.122 (3)	
C20	0.1672 (6)	0.6226 (5)	0.7861 (3)	0.127 (2)	
C21	0.1826 (5)	0.6855 (4)	0.7056 (3)	0.113 (2)	
C22	0.2493 (4)	0.7587 (4)	0.5465 (3)	0.0929 (6)	
C23	0.2813 (4)	0.8062 (4)	0.4627 (3)	0.0929 (6)	
C24	0.3134 (4)	0.9242 (4)	0.4648 (3)	0.0929 (6)	
C25	0.3459 (4)	0.9759 (4)	0.3907 (3)	0.0929 (6)	
C26	0.3471 (4)	0.9074 (4)	0.3098 (3)	0.0929 (6)	
C27	0.3138 (4)	0.7870 (4)	0.3061 (3)	0.0929 (6)	
C28	0.2819 (4)	0.7383 (4)	0.3821 (3)	0.0929 (6)	
C29	0.4144 (4)	1.0752 (3)	0.2382 (2)	0.0812 (16)	
C30	0.4472 (3)	1.1038 (3)	0.1500 (2)	0.0691 (12)	
C31	0.3727 (3)	1.1294 (3)	0.0736 (2)	0.0750 (14)	
C32A	0.4142 (19)	1.1840 (16)	−0.0775 (8)	0.098 (3)	0.705 (15)
C33A	0.4217 (15)	1.3152 (11)	−0.0709 (7)	0.141 (5)	0.705 (15)
C34A	0.3135 (19)	1.3541 (19)	−0.0881 (16)	0.205 (9)	0.705 (15)
C33B	0.343 (3)	1.278 (2)	−0.074 (2)	0.141 (5)	0.295 (15)
C34B	0.379 (6)	1.384 (3)	−0.094 (4)	0.205 (9)	0.295 (15)
C32B	0.440 (5)	1.195 (5)	−0.076 (2)	0.098 (3)	0.295 (15)
H3A	0.80520	0.54850	0.85160	0.1770*	
H3B	0.71260	0.64010	0.85230	0.1770*	
H1A	0.56070	0.49750	0.61150	0.1600*	
H1B	0.47860	0.59630	0.62200	0.1600*	
H2A	0.49390	0.58410	0.77570	0.1700*	
H2B	0.44330	0.45630	0.72220	0.1700*	
H10	0.63680	0.86200	0.28980	0.1320*	
H11	0.58630	0.76870	0.40960	0.1520*	
H12A	1.03440	0.93890	0.25500	0.1160*	
H12B	1.02340	1.05810	0.31030	0.1160*	
H14	0.79060	1.07480	0.12090	0.0960*	
H15A	0.77360	1.15440	−0.04370	0.1080*	0.691 (10)
H15B	0.89010	1.12750	−0.08910	0.1080*	0.691 (10)
H16A	0.92450	1.34780	0.02420	0.1800*	0.691 (10)
H17A	0.95080	1.28890	−0.15590	0.2710*	0.691 (10)
H17B	0.98890	1.41980	−0.09920	0.2710*	0.691 (10)
H4A	0.84570	0.68580	0.75060	0.1630*	
H4B	0.80600	0.55680	0.69810	0.1630*	
H5A	0.85650	0.72520	0.60660	0.1100*	0.576 (7)
H7	0.96150	0.80980	0.52080	0.1810*	
H8	1.01810	0.90640	0.40650	0.1380*	
H5B	0.59290	0.67730	0.51930	0.1100*	0.424 (7)
H15C	0.79010	1.22070	0.00380	0.1080*	0.309 (10)
H15D	0.80820	1.12550	−0.07200	0.1080*	0.309 (10)
H16B	1.00100	1.28700	−0.09410	0.1800*	0.309 (10)
H17C	0.78530	1.33640	−0.01440	0.2710*	0.309 (10)
H17D	0.86780	1.43510	−0.06060	0.2710*	0.309 (10)
H19A	0.03400	0.37320	0.67800	0.1470*	
H20A	0.25290	0.61180	0.81630	0.1520*	
H20B	0.13580	0.67260	0.82970	0.1520*	
H19B	0.18520	0.41180	0.71510	0.1470*	
H18A	0.15380	0.44570	0.56980	0.1480*	
H18B	0.03120	0.50150	0.57790	0.1480*	
H24	0.31340	0.97260	0.51850	0.1110*	
H25	0.36730	1.05750	0.39530	0.1110*	
H27	0.31270	0.73820	0.25240	0.1110*	
H28	0.26010	0.65680	0.37820	0.1110*	
H29A	0.48860	1.10690	0.28860	0.0970*	
H29B	0.34100	1.11010	0.24940	0.0970*	
H31	0.28370	1.13050	0.06390	0.0900*	
H32A	0.32600	1.14320	−0.10620	0.1170*	0.705 (15)
H32B	0.47310	1.16050	−0.11540	0.1170*	0.705 (15)
H33A	0.50200	1.36700	−0.05490	0.1690*	0.705 (15)
H34A	0.23380	1.30140	−0.10410	0.2460*	0.705 (15)
H34B	0.31550	1.43460	−0.08450	0.2460*	0.705 (15)
H21A	0.10640	0.72010	0.68740	0.1350*	
H21B	0.25860	0.74890	0.72340	0.1350*	
H22	0.25320	0.81040	0.59930	0.1110*	
H32C	0.40690	1.12990	−0.12530	0.1170*	0.295 (15)
H32D	0.52390	1.23630	−0.08300	0.1170*	0.295 (15)
H33B	0.26240	1.25350	−0.05920	0.1690*	0.295 (15)
H34C	0.46010	1.40690	−0.10910	0.2460*	0.295 (15)
H34D	0.32210	1.43730	−0.09410	0.2460*	0.295 (15)

### Atomic displacement parameters (Å^2^)

**Table:**

	*U*^11^	*U*^22^	*U*^33^	*U*^12^	*U*^13^	*U*^23^
O1	0.168 (4)	0.110 (3)	0.171 (3)	0.029 (3)	0.055 (3)	0.055 (3)
O2	0.075 (2)	0.138 (3)	0.0951 (19)	0.0063 (17)	0.0098 (15)	0.0294 (19)
N1	0.160 (4)	0.103 (3)	0.142 (4)	0.044 (3)	0.072 (3)	0.036 (3)
N2A	0.091 (6)	0.091 (4)	0.109 (6)	0.028 (5)	0.032 (4)	0.017 (5)
N3	0.057 (2)	0.125 (3)	0.129 (3)	0.011 (2)	0.024 (2)	0.017 (2)
N4	0.060 (2)	0.122 (3)	0.138 (3)	0.016 (2)	0.039 (2)	0.020 (3)
N5	0.060 (2)	0.078 (2)	0.107 (2)	0.0106 (16)	0.0241 (18)	0.0067 (18)
C1	0.144 (5)	0.132 (5)	0.143 (4)	0.038 (4)	0.051 (4)	0.050 (4)
C2	0.140 (5)	0.142 (5)	0.164 (5)	0.038 (4)	0.054 (4)	0.064 (4)
C3	0.173 (6)	0.102 (4)	0.171 (5)	0.017 (4)	0.040 (4)	0.042 (4)
C4	0.149 (5)	0.091 (4)	0.179 (5)	0.018 (3)	0.056 (4)	0.038 (4)
C5A	0.072 (5)	0.087 (5)	0.103 (6)	0.012 (5)	0.001 (4)	−0.014 (5)
C6	0.324 (12)	0.073 (4)	0.089 (4)	0.056 (5)	0.089 (6)	0.019 (3)
C7	0.274 (10)	0.095 (4)	0.087 (4)	0.059 (5)	0.019 (5)	0.019 (3)
C8	0.134 (4)	0.099 (3)	0.096 (3)	0.023 (3)	−0.005 (3)	−0.004 (3)
C9	0.102 (4)	0.092 (3)	0.076 (3)	0.017 (3)	0.013 (2)	0.000 (2)
C10	0.115 (4)	0.123 (4)	0.092 (3)	0.017 (3)	0.031 (3)	0.007 (3)
C11	0.178 (6)	0.104 (4)	0.108 (4)	0.017 (4)	0.069 (4)	0.012 (3)
C12	0.069 (3)	0.104 (3)	0.107 (3)	0.013 (2)	0.009 (2)	−0.002 (3)
C13	0.065 (3)	0.079 (3)	0.091 (3)	0.012 (2)	0.016 (2)	−0.004 (2)
C14	0.055 (2)	0.085 (3)	0.102 (3)	0.010 (2)	0.025 (2)	0.010 (2)
C15A	0.079 (5)	0.088 (7)	0.109 (5)	0.016 (3)	0.036 (4)	0.013 (5)
C16A	0.182 (8)	0.171 (9)	0.089 (10)	0.013 (7)	0.012 (8)	0.052 (9)
C17A	0.183 (12)	0.242 (12)	0.222 (12)	−0.052 (9)	−0.013 (8)	0.156 (11)
C5B	0.072 (5)	0.087 (5)	0.103 (6)	0.012 (5)	0.001 (4)	−0.014 (5)
C15B	0.079 (5)	0.088 (7)	0.109 (5)	0.016 (3)	0.036 (4)	0.013 (5)
C16B	0.182 (8)	0.171 (9)	0.089 (10)	0.013 (7)	0.012 (8)	0.052 (9)
C17B	0.183 (12)	0.242 (12)	0.222 (12)	−0.052 (9)	−0.013 (8)	0.156 (11)
N2B	0.091 (6)	0.091 (4)	0.109 (6)	0.028 (5)	0.032 (4)	0.017 (5)
O3	0.215 (4)	0.118 (3)	0.116 (3)	0.025 (3)	0.052 (3)	0.039 (2)
O4	0.109 (2)	0.0753 (18)	0.0840 (16)	0.0220 (15)	0.0296 (14)	0.0079 (13)
N6	0.116 (2)	0.098 (2)	0.1014 (18)	0.0198 (16)	0.0324 (15)	0.0202 (15)
N7	0.116 (2)	0.098 (2)	0.1014 (18)	0.0198 (16)	0.0324 (15)	0.0202 (15)
N8	0.057 (2)	0.132 (3)	0.104 (3)	0.0254 (19)	0.0182 (17)	0.025 (2)
N9	0.057 (2)	0.145 (3)	0.104 (3)	0.020 (2)	0.0252 (18)	0.026 (2)
N10	0.058 (2)	0.096 (2)	0.089 (2)	0.0163 (16)	0.0205 (16)	0.0189 (17)
C18	0.174 (5)	0.084 (3)	0.105 (3)	0.015 (3)	0.021 (3)	0.017 (3)
C19	0.151 (5)	0.100 (4)	0.121 (4)	0.019 (3)	0.044 (3)	0.022 (3)
C20	0.172 (5)	0.111 (4)	0.099 (3)	0.013 (4)	0.044 (3)	0.020 (3)
C21	0.158 (5)	0.092 (3)	0.087 (3)	0.028 (3)	0.017 (3)	0.015 (3)
C22	0.1033 (12)	0.0795 (9)	0.0963 (11)	0.0200 (9)	0.0218 (9)	0.0087 (7)
C23	0.1033 (12)	0.0795 (9)	0.0963 (11)	0.0200 (9)	0.0218 (9)	0.0087 (7)
C24	0.1033 (12)	0.0795 (9)	0.0963 (11)	0.0200 (9)	0.0218 (9)	0.0087 (7)
C25	0.1033 (12)	0.0795 (9)	0.0963 (11)	0.0200 (9)	0.0218 (9)	0.0087 (7)
C26	0.1033 (12)	0.0795 (9)	0.0963 (11)	0.0200 (9)	0.0218 (9)	0.0087 (7)
C27	0.1033 (12)	0.0795 (9)	0.0963 (11)	0.0200 (9)	0.0218 (9)	0.0087 (7)
C28	0.1033 (12)	0.0795 (9)	0.0963 (11)	0.0200 (9)	0.0218 (9)	0.0087 (7)
C29	0.082 (3)	0.078 (3)	0.081 (2)	0.018 (2)	0.013 (2)	0.002 (2)
C30	0.059 (2)	0.070 (2)	0.080 (2)	0.0165 (18)	0.0180 (18)	0.0055 (18)
C31	0.053 (2)	0.093 (3)	0.087 (2)	0.0212 (19)	0.025 (2)	0.019 (2)
C32A	0.082 (8)	0.124 (6)	0.093 (3)	0.019 (6)	0.027 (3)	0.031 (3)
C33A	0.153 (12)	0.128 (9)	0.151 (6)	0.015 (8)	0.038 (8)	0.073 (6)
C34A	0.26 (2)	0.202 (15)	0.223 (10)	0.107 (14)	0.117 (16)	0.121 (10)
C33B	0.153 (12)	0.128 (9)	0.151 (6)	0.015 (8)	0.038 (8)	0.073 (6)
C34B	0.26 (2)	0.202 (15)	0.223 (10)	0.107 (14)	0.117 (16)	0.121 (10)
C32B	0.082 (8)	0.124 (6)	0.093 (3)	0.019 (6)	0.027 (3)	0.031 (3)

### Geometric parameters (Å, º)

**Table:**

O1—C2	1.405 (8)	C8—H8	0.9300
O1—C3	1.402 (8)	C10—H10	0.9300
O2—C9	1.363 (5)	C11—H11	0.9300
O2—C12	1.404 (6)	C12—H12A	0.9700
O3—C20	1.377 (8)	C12—H12B	0.9700
O3—C19	1.390 (8)	C14—H14	0.9300
O4—C26	1.364 (5)	C15A—H15B	0.9700
O4—C29	1.427 (4)	C15A—H15A	0.9700
N1—N2A	1.472 (10)	C15B—H15D	0.9600
N1—C1	1.426 (9)	C15B—H15C	0.9700
N1—C4	1.435 (8)	C16A—H16A	0.9300
N1—N2B	1.384 (13)	C16B—H16B	0.9300
N2A—C5A	1.277 (12)	C17A—H17A	0.9300
N2B—C5B	1.244 (16)	C17A—H17B	0.9300
N3—C13	1.343 (6)	C17B—H17D	0.9400
N3—N4	1.302 (6)	C17B—H17C	0.9300
N4—N5	1.337 (5)	C18—C19	1.475 (7)
N5—C14	1.329 (5)	C20—C21	1.485 (7)
N5—C15B	1.47 (4)	C22—C23	1.486 (6)
N5—C15A	1.460 (15)	C23—C24	1.353 (7)
N6—N7	1.396 (5)	C23—C28	1.369 (6)
N6—C18	1.413 (6)	C24—C25	1.379 (6)
N6—C21	1.421 (6)	C25—C26	1.378 (6)
N7—C22	1.237 (6)	C26—C27	1.378 (7)
N8—N9	1.307 (6)	C27—C28	1.386 (6)
N8—C30	1.358 (5)	C29—C30	1.474 (4)
N9—N10	1.336 (5)	C30—C31	1.349 (4)
N10—C31	1.324 (4)	C32A—C33A	1.51 (2)
N10—C32A	1.460 (13)	C32B—C33B	1.53 (6)
N10—C32B	1.48 (4)	C33A—C34A	1.30 (3)
C1—C2	1.492 (8)	C33B—C34B	1.31 (4)
C3—C4	1.494 (8)	C18—H18A	0.9700
C5A—C6	1.455 (10)	C18—H18B	0.9700
C5B—C6	1.574 (16)	C19—H19A	0.9700
C6—C11	1.390 (11)	C19—H19B	0.9700
C6—C7	1.360 (14)	C20—H20A	0.9700
C7—C8	1.343 (8)	C20—H20B	0.9700
C8—C9	1.389 (7)	C21—H21A	0.9700
C9—C10	1.380 (8)	C21—H21B	0.9700
C10—C11	1.362 (7)	C22—H22	0.9300
C12—C13	1.468 (6)	C24—H24	0.9300
C13—C14	1.350 (6)	C25—H25	0.9300
C15A—C16A	1.48 (3)	C27—H27	0.9300
C15B—C16B	1.51 (6)	C28—H28	0.9300
C16A—C17A	1.325 (19)	C29—H29A	0.9700
C16B—C17B	1.35 (6)	C29—H29B	0.9700
C1—H1B	0.9700	C31—H31	0.9300
C1—H1A	0.9700	C32A—H32A	0.9700
C2—H2A	0.9700	C32A—H32B	0.9700
C2—H2B	0.9700	C32B—H32C	0.9700
C3—H3A	0.9700	C32B—H32D	0.9600
C3—H3B	0.9700	C33A—H33A	0.9300
C4—H4B	0.9700	C33B—H33B	0.9300
C4—H4A	0.9700	C34A—H34A	0.9300
C5A—H5A	0.9300	C34A—H34B	0.9300
C5B—H5B	0.9300	C34B—H34C	0.9300
C7—H7	0.9300	C34B—H34D	0.9400
			
C2—O1—C3	109.9 (4)	H15A—C15A—H15B	109.00
C9—O2—C12	119.7 (4)	C16A—C15A—H15A	110.00
C19—O3—C20	114.1 (5)	C16B—C15B—H15D	108.00
C26—O4—C29	117.3 (3)	N5—C15B—H15D	108.00
N2A—N1—C4	128.8 (6)	N5—C15B—H15C	107.00
C1—N1—C4	113.2 (5)	H15C—C15B—H15D	107.00
N2B—N1—C4	94.6 (6)	C16B—C15B—H15C	107.00
N2B—N1—C1	135.3 (6)	C17A—C16A—H16A	121.00
N2A—N1—C1	101.4 (5)	C15A—C16A—H16A	121.00
N1—N2A—C5A	112.4 (8)	C17B—C16B—H16B	134.00
N1—N2B—C5B	110.8 (11)	C15B—C16B—H16B	134.00
N4—N3—C13	109.1 (3)	H17A—C17A—H17B	120.00
N3—N4—N5	107.0 (3)	C16A—C17A—H17B	120.00
N4—N5—C15B	125.8 (17)	C16A—C17A—H17A	120.00
C14—N5—C15A	131.4 (8)	H17C—C17B—H17D	119.00
N4—N5—C14	110.4 (3)	C16B—C17B—H17D	120.00
N4—N5—C15A	118.0 (8)	C16B—C17B—H17C	121.00
C14—N5—C15B	123.4 (17)	N6—C18—C19	113.3 (4)
C18—N6—C21	117.0 (4)	O3—C19—C18	114.8 (5)
N7—N6—C21	121.4 (3)	O3—C20—C21	115.9 (4)
N7—N6—C18	110.7 (4)	N6—C21—C20	112.1 (4)
N6—N7—C22	119.6 (3)	N7—C22—C23	121.1 (4)
N9—N8—C30	109.5 (3)	C22—C23—C24	118.9 (4)
N8—N9—N10	106.7 (3)	C22—C23—C28	124.4 (4)
N9—N10—C31	110.3 (3)	C24—C23—C28	116.7 (4)
N9—N10—C32B	113 (2)	C23—C24—C25	122.9 (4)
C31—N10—C32A	127.0 (8)	C24—C25—C26	120.4 (4)
N9—N10—C32A	122.7 (9)	O4—C26—C25	124.6 (4)
C31—N10—C32B	137 (2)	O4—C26—C27	117.8 (4)
N1—C1—C2	112.2 (5)	C25—C26—C27	117.6 (4)
O1—C2—C1	112.0 (5)	C26—C27—C28	120.3 (4)
O1—C3—C4	112.1 (5)	C23—C28—C27	122.2 (4)
N1—C4—C3	111.1 (6)	O4—C29—C30	109.1 (3)
N2A—C5A—C6	110.1 (9)	N8—C30—C29	122.4 (3)
N2B—C5B—C6	110.5 (11)	N8—C30—C31	106.7 (3)
C5B—C6—C7	144.9 (8)	C29—C30—C31	130.9 (3)
C5A—C6—C7	106.1 (7)	N10—C31—C30	106.8 (3)
C7—C6—C11	115.1 (6)	N10—C32A—C33A	109.2 (10)
C5B—C6—C11	100.0 (9)	N10—C32B—C33B	104 (3)
C5A—C6—C11	138.8 (9)	C32A—C33A—C34A	118.4 (16)
C6—C7—C8	125.5 (7)	C32B—C33B—C34B	117 (4)
C7—C8—C9	118.2 (6)	N6—C18—H18A	109.00
O2—C9—C8	124.4 (5)	N6—C18—H18B	109.00
C8—C9—C10	119.2 (4)	C19—C18—H18A	109.00
O2—C9—C10	116.4 (4)	C19—C18—H18B	109.00
C9—C10—C11	119.8 (5)	H18A—C18—H18B	108.00
C6—C11—C10	122.3 (7)	O3—C19—H19A	109.00
O2—C12—C13	108.5 (4)	O3—C19—H19B	109.00
N3—C13—C12	120.9 (4)	C18—C19—H19A	109.00
C12—C13—C14	131.0 (4)	C18—C19—H19B	109.00
N3—C13—C14	108.1 (4)	H19A—C19—H19B	108.00
N5—C14—C13	105.4 (4)	O3—C20—H20A	108.00
N5—C15A—C16A	106.9 (11)	O3—C20—H20B	108.00
N5—C15B—C16B	118 (3)	C21—C20—H20A	108.00
C15A—C16A—C17A	118.9 (12)	C21—C20—H20B	108.00
C15B—C16B—C17B	92 (4)	H20A—C20—H20B	107.00
N1—C1—H1A	109.00	N6—C21—H21A	109.00
N1—C1—H1B	109.00	N6—C21—H21B	109.00
C2—C1—H1A	109.00	C20—C21—H21A	109.00
C2—C1—H1B	109.00	C20—C21—H21B	109.00
H1A—C1—H1B	108.00	H21A—C21—H21B	108.00
H2A—C2—H2B	108.00	N7—C22—H22	119.00
O1—C2—H2A	109.00	C23—C22—H22	119.00
C1—C2—H2B	109.00	C23—C24—H24	119.00
C1—C2—H2A	109.00	C25—C24—H24	119.00
O1—C2—H2B	109.00	C24—C25—H25	120.00
C4—C3—H3A	109.00	C26—C25—H25	120.00
O1—C3—H3B	109.00	C26—C27—H27	120.00
O1—C3—H3A	109.00	C28—C27—H27	120.00
H3A—C3—H3B	108.00	C23—C28—H28	119.00
C4—C3—H3B	109.00	C27—C28—H28	119.00
H4A—C4—H4B	108.00	O4—C29—H29A	110.00
C3—C4—H4B	109.00	O4—C29—H29B	110.00
N1—C4—H4A	109.00	C30—C29—H29A	110.00
N1—C4—H4B	109.00	C30—C29—H29B	110.00
C3—C4—H4A	109.00	H29A—C29—H29B	108.00
C6—C5A—H5A	125.00	N10—C31—H31	127.00
N2A—C5A—H5A	125.00	C30—C31—H31	127.00
N2B—C5B—H5B	125.00	N10—C32A—H32A	110.00
C6—C5B—H5B	125.00	N10—C32A—H32B	110.00
C6—C7—H7	117.00	C33A—C32A—H32A	110.00
C8—C7—H7	117.00	C33A—C32A—H32B	110.00
C7—C8—H8	121.00	H32A—C32A—H32B	108.00
C9—C8—H8	121.00	C33B—C32B—H32C	111.00
C11—C10—H10	120.00	C33B—C32B—H32D	111.00
C9—C10—H10	120.00	H32C—C32B—H32D	110.00
C10—C11—H11	119.00	N10—C32B—H32D	111.00
C6—C11—H11	119.00	N10—C32B—H32C	111.00
H12A—C12—H12B	108.00	C32A—C33A—H33A	121.00
C13—C12—H12B	110.00	C34A—C33A—H33A	121.00
O2—C12—H12A	110.00	C32B—C33B—H33B	121.00
O2—C12—H12B	110.00	C34B—C33B—H33B	122.00
C13—C12—H12A	110.00	C33A—C34A—H34A	120.00
N5—C14—H14	127.00	C33A—C34A—H34B	120.00
C13—C14—H14	127.00	H34A—C34A—H34B	120.00
N5—C15A—H15A	110.00	C33B—C34B—H34C	121.00
C16A—C15A—H15B	110.00	C33B—C34B—H34D	120.00
N5—C15A—H15B	110.00	H34C—C34B—H34D	119.00
			
C3—O1—C2—C1	58.0 (6)	N9—N10—C32A—C33A	−94.1 (14)
C2—O1—C3—C4	−59.2 (7)	C31—N10—C32A—C33A	86.5 (14)
C12—O2—C9—C10	178.0 (4)	N1—C1—C2—O1	−52.9 (7)
C9—O2—C12—C13	−180.0 (4)	O1—C3—C4—N1	54.6 (7)
C12—O2—C9—C8	−1.0 (7)	N2A—C5A—C6—C7	176.6 (7)
C19—O3—C20—C21	48.3 (7)	N2A—C5A—C6—C11	−0.8 (13)
C20—O3—C19—C18	−48.2 (7)	C7—C6—C11—C10	0.0 (9)
C26—O4—C29—C30	−179.5 (3)	C5A—C6—C11—C10	177.2 (8)
C29—O4—C26—C27	179.3 (4)	C11—C6—C7—C8	0.9 (10)
C29—O4—C26—C25	−2.1 (6)	C5A—C6—C7—C8	−177.3 (6)
N2A—N1—C4—C3	−177.2 (6)	C6—C7—C8—C9	−1.1 (9)
C1—N1—N2A—C5A	−150.7 (7)	C7—C8—C9—C10	0.5 (7)
C1—N1—C4—C3	−48.8 (6)	C7—C8—C9—O2	179.5 (5)
N2A—N1—C1—C2	−170.2 (6)	C8—C9—C10—C11	0.3 (7)
C4—N1—N2A—C5A	−18.0 (11)	O2—C9—C10—C11	−178.8 (5)
C4—N1—C1—C2	48.3 (7)	C9—C10—C11—C6	−0.5 (8)
N1—N2A—C5A—C6	−175.1 (6)	O2—C12—C13—N3	−165.0 (4)
C13—N3—N4—N5	−0.4 (5)	O2—C12—C13—C14	14.3 (6)
N4—N3—C13—C14	0.2 (5)	N3—C13—C14—N5	0.1 (5)
N4—N3—C13—C12	179.7 (4)	C12—C13—C14—N5	−179.3 (4)
N3—N4—N5—C15A	−174.1 (8)	N5—C15A—C16A—C17A	135.4 (15)
N3—N4—N5—C14	0.5 (5)	N6—C18—C19—O3	44.6 (7)
C14—N5—C15A—C16A	118.4 (12)	O3—C20—C21—N6	−44.0 (7)
C15A—N5—C14—C13	173.3 (9)	N7—C22—C23—C28	−1.6 (7)
N4—N5—C15A—C16A	−68.3 (14)	N7—C22—C23—C24	179.0 (4)
N4—N5—C14—C13	−0.4 (4)	C22—C23—C28—C27	−179.3 (4)
C21—N6—C18—C19	−42.0 (7)	C22—C23—C24—C25	179.3 (4)
C18—N6—C21—C20	41.2 (6)	C24—C23—C28—C27	0.1 (7)
N7—N6—C18—C19	173.2 (5)	C28—C23—C24—C25	−0.2 (7)
C18—N6—N7—C22	157.9 (5)	C23—C24—C25—C26	−0.1 (7)
C21—N6—N7—C22	15.0 (7)	C24—C25—C26—C27	0.6 (7)
N7—N6—C21—C20	−178.0 (4)	C24—C25—C26—O4	−178.0 (4)
N6—N7—C22—C23	175.2 (4)	C25—C26—C27—C28	−0.7 (6)
C30—N8—N9—N10	−0.3 (4)	O4—C26—C27—C28	178.0 (4)
N9—N8—C30—C31	0.3 (4)	C26—C27—C28—C23	0.4 (7)
N9—N8—C30—C29	−177.8 (3)	O4—C29—C30—N8	−88.2 (4)
N8—N9—N10—C32A	−179.3 (9)	O4—C29—C30—C31	94.2 (5)
N8—N9—N10—C31	0.2 (4)	N8—C30—C31—N10	−0.1 (4)
C32A—N10—C31—C30	179.5 (9)	C29—C30—C31—N10	177.8 (4)
N9—N10—C31—C30	−0.1 (4)	N10—C32A—C33A—C34A	−109.6 (17)

### Hydrogen-bond geometry (Å, º)

**Table:**

*D*—H···*A*	*D*—H	H···*A*	*D*···*A*	*D*—H···*A*
C14—H14···N8	0.93	2.49	3.385 (6)	162
C15*A*—H15*B*···N3^i^	0.97	2.59	3.355 (18)	136
C31—H31···N3^ii^	0.93	2.54	3.323 (5)	141
C31—H31···N4^ii^	0.93	2.40	3.326 (5)	171
C24—H24···*Cg*3^iii^	0.93	2.92	3.710 (5)	144

Symmetry codes: (i) −*x*+2, −*y*+2, −*z*; (ii) *x*−1, *y*, *z*; (iii) −*x*+1, −*y*+2, −*z*+1.
